# Dynamical modeling of TNF-α, IL-6, and IL-10 interactions in stroke-induced inflammation

**DOI:** 10.1371/journal.pone.0339178

**Published:** 2026-01-20

**Authors:** Fozia Ali M. Arishi, Azmin Sham Rambely, Fatimah Abdul Razak

**Affiliations:** 1 Department of Mathematical Sciences, Faculty of Science and Technology, Universiti Kebangsaan Malaysia, Bangi, Selangor, Malaysia; 2 Department of Mathematics, College of Science, Jazan University, Jazan, Kingdom of Saudi Arabia; University of Rijeka Faculty of Health Studies: Sveuciliste u Rijeci Fakultet zdravstvenih studija, CROATIA

## Abstract

In acute stroke, dysregulated cytokine interactions drive secondary injury, yet bidirectional feedback mechanisms between pro-inflammatory mediators (TNF-α, IL-6) and the anti-inflammatory mediator IL-10 remain poorly quantified. We developed a systems biology model using nonlinear ordinary differential equations (ODEs) to resolve these dynamics, incorporating Nuclear Factor kappa-light-chain-enhancer of activated B cells (NF-κB)-mediated cross-activation, delayed IL-10 induction via a Hill function, and empirical parameterization from stroke data. Mathematical analysis revealed bistable inflammatory states via bifurcation theory, mechanistically explaining divergent inflammatory trajectory. Steady-state and stability analyses identified a critical IL-10 suppression threshold ($\gamma_{T\text{1}\text{0}}\approx \text{0}.\text{0}\text{6}$ hr ⁻ ¹·nM ⁻ ¹) governing transitions between pro-inflammatory dominance and resolution phases. The model replicated experimentally observed cytokine dynamics, including TNF-α/IL-6 peaks (6–24 hours) and delayed IL-10 elevation (48 hours). Global sensitivity analysis highlighted IL-10 production ($k_{\text{1}\text{0}T}$) and TNF-α suppression ($\gamma_{T\text{1}\text{0}}$) as key control parameters. Simulations predicted that IL-10 augmentation accelerates resolution, while TNF-α inhibition attenuates IL-10 induction, potentially compromising long-term recovery. By integrating dynamical systems theory with translational immunology, this model provides a mechanistic basis for optimizing immunomodulatory therapies in stroke and related inflammatory pathologies.

## 1. Introduction

Inflammatory processes are central to the pathophysiology of stroke, where dysregulated cytokine signaling exacerbates tissue damage and impedes recovery [1,2]. Tumor necrosis factor-alpha (TNF-α) and interleukin-6 (IL-6) drive pro-inflammatory cascades that disrupt the blood-brain barrier, amplify oxidative stress, and promote neuronal apoptosis, while interleukin-10 (IL-10) counteracts these effects by suppressing immune activation and facilitating tissue repair [3,4]. Clinical studies demonstrate that imbalances in this triad, persistent TNF-α/IL-6 levels with insufficient IL-10, correlate with larger infarct volumes and poorer neurological outcomes [5, 6]. However, the temporal dynamics governing their interactions, particularly how feedback loops between pro- and anti-inflammatory cytokines resolve or perpetuate inflammation, remain poorly quantified [7].

Existing models often neglect these bidirectional regulatory mechanisms, limiting their utility for predicting therapeutic outcomes [8]. Furthermore, while interleukin-1 (IL-1) is a critical mediator in post-stroke inflammation, our model focuses on the TNF-α/IL-6/IL-10 axis to establish a foundational understanding of their core feedback loops.

Hanging approaches face limitations in capturing real-time, system-level cytokine interactions due to the spatial heterogeneity of neuroinflammation and ethical constraints on repeated sampling in human studies [9]. While computational models have explored isolated aspects of post-stroke inflammation, such as TNF-α-mediated microglial activation [10] or IL-6’s dual neurotoxic/neuroprotective roles [11], no study has yet integrated TNF-α, IL-6, and IL-10 into a unified mathematical model. This gap is critical, as IL-6 not only amplifies TNF-α production via Nuclear Factor kappa-light-chain-enhancer of activated B cells (NF-κB) signalling, but also induces IL-10 synthesis to self-limit inflammation [12]. NF-κB is a transcription factor complex that acts as a central regulator of inflammatory responses; it is activated by upstream cytokine signaling (e.g., TNF-α and IL-6) and, in turn, promotes the transcription of pro-inflammatory genes, including TNF-α and IL-6 themselves. This creates a positive feedback loop that sustains inflammation unless counter-regulated by anti-inflammatory mediators like IL-10 [13]. Existing models often neglect these bidirectional regulatory mechanisms, limiting their utility for predicting therapeutic outcomes [8].

In this study, a system of ordinary differential equations (ODEs) is developed to quantify the dynamic interactions between TNF-α, IL-6, and IL-10 in acute stroke. The model explicitly incorporates nonlinear feedback (IL-10 suppression of TNF-α/IL-6), cross-activation (NF-κB-mediated TNF-α/IL-6 synergy), and empirical parameterization from clinical stroke data. Our objectives were threefold: capture general cytokine response patterns observed in clinical stroke studies, including early TNF-α/IL-6 peaks and delayed IL-10 elevation; to predict the therapeutic effects of IL-10 augmentation and TNF-α inhibition on inflammation resolution; and to identify critical parameters governing transitions between pro- and anti-inflammatory states. By bridging mechanistic immunology with dynamical systems theory, this framework provides a predictive tool for optimizing immunomodulatory strategies in stroke and related inflammatory pathologies.

## 2. Materials and methods

To quantitatively characterize the dynamic interplay among TNF-α, IL-6, and IL-10 during acute post-stroke inflammation, a system of ordinary differential equations (ODEs) was developed grounded in experimental evidence [11–14] and validated clinical observations [5,6]. This modeling framework integrates both pro-inflammatory and anti-inflammatory signaling cascades and captures the nonlinear feedback mechanisms that underlie immune regulation in stroke.

### 2.1. Model formulation and biological assumptions

The model is constructed around three core variables representing the concentrations of key cytokines involved in stroke-induced inflammation. The concentration of TNF-α,$\;C_{T}$, a principal pro-inflammatory cytokine known to initiate and amplify immune responses following ischemic injury. $C_{\text{6}}$ corresponds to IL-6, a pleiotropic cytokine that plays dual roles in neuroinflammation, contributing both to neurotoxic damage and neuroprotective repair mechanisms. Finally, $C_{\text{1}\text{0}}$ represents IL-10, an anti-inflammatory mediator that suppresses immune activation and promotes tissue healing during the resolution phase of inflammation.

Biologically, TNF-α and IL-6 are primarily produced by activated microglia and infiltrating macrophages following ischemic injury [11,15], while IL-10 is secreted in response to inflammatory stimuli by regulatory T cells and astrocytes [6,12]. The model assumes cytokine degradation follows first-order kinetics, consistent with existing inflammation models [16,17].

**Fig 1:**
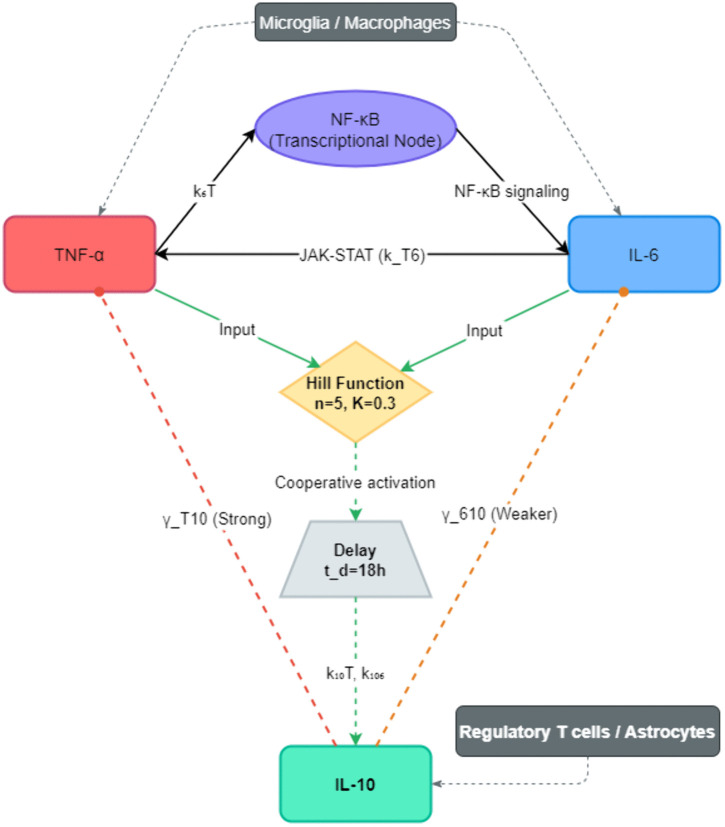
Dynamical network of TNF-α, IL-6, and IL-10 interactions. The diagram depicts the core structure of the computational model, including the pro-inflammatory amplification loop between TNF-α and IL-6 (blue arrows), the delayed induction of IL-10 via a Hill function (green pathway), and the feedback suppression of TNF-α and IL-6 by IL-10 (red, dashed lines). Cellular sources are indicated. Model parameters (e.g., $\gamma_{T\text{1}\text{0}}$, $k_{\text{6}T}$) correspond to those defined in Equations 1–3.

To ensure biological fidelity, the model incorporates well-established cytokine mechanisms governing stroke-induced inflammation. Each regulatory term is grounded in experimentally validated feedback or feedforward interactions, which are summarized conceptually in Fig 1. These interactions, illustrated in Fig 1, highlight a bidirectional regulatory loop wherein TNF-α activates IL-6 production via NF-κB (pathway $k_{T\text{6}}$), and IL-6 in turn reinforces TNF-α expression, forming a self-amplifying pro-inflammatory circuit. The model also incorporates delayed IL-10 induction and its inhibitory effects on both TNF-α and IL-6, capturing the dynamic balance between immune activation and resolution.

#### 2.2.1. Bidirectional pro-inflammatory signaling.

TNF-α, a primary early mediator of neuroinflammation, triggers the synthesis of IL-6 by activating the NF-κB transcriptional pathway. This interaction is modeled through the rate constant $k_{\text{6}T}$, representing IL-6 production in response to TNF-α. Conversely, IL-6 enhances TNF-α expression via the coefficient $k_{T\text{6}}$, thereby establishing a bidirectional, self-amplifying loop. This mutual reinforcement is well-documented in both in vitro and in vivo stroke and endotoxemia models, where NF-κB signaling serves as a convergence node for pro-inflammatory cytokine co-expression [13,18]. The model captures this synergy as a key driver of the rapid inflammatory escalation observed in the early hours post-stroke. In this framework, the NF-κB pathway is used as a conceptual node representing the net effect of convergent pro-inflammatory signaling that leads to the coordinated expression of TNF-α and IL-6. It is important to note that while TNF-α is a primary activator of NF-κB, IL-6 signals primarily through the JAK-STAT pathway [12]; the cross-activation terms thus capture their observed synergy in vivo rather than a single literal signaling cascade.

#### 2.2.2. Anti-inflammatory feedback through IL-10 suppression.

Interleukin-10 (IL-10) acts as a pivotal immunoregulatory checkpoint, mitigating the inflammatory cascade by suppressing TNF-α and IL-6 production. This suppression is encoded in the model via nonlinear inhibitory terms $\gamma_{T\text{1}\text{0}}C_{\text{1}\text{0}}C_{T}$ and $\gamma_{\text{6}\text{1}\text{0}}C_{\text{1}\text{0}}C_{\text{6}}$, reflecting concentration-dependent inhibition. Mechanistically, IL-10 exerts its effects by activating the Signal Transducer and Activator of Transcription 3 (STAT3), a key intracellular signaling protein that downregulates inflammatory gene transcription and limits cytokine amplification [4,12]. Although STAT3 is not explicitly shown in Fig 1, its function is implicitly represented through the IL-10–mediated inhibitory pathways. These suppression pathways are essential for transitioning from a pro-inflammatory to a resolution phase and preventing excessive neuronal injury. Notably, the strength of suppression differs: TNF-α inhibition is typically more pronounced than IL-6, consistent with experimental findings and parameter assignments in the model [5,18,19].

#### 2.2.3. Delayed induction of IL-10 and inflammation resolution.

Unlike TNF-α and IL-6, which surge rapidly post-insult, IL-10 expression is temporally delayed, providing a window of early immune activation before feedback suppression initiates. The model represents this via additive production terms $k_{T\text{6}}C_{\text{6}}$ and $k_{\text{6}T}C_{T}$, indicating that IL-10 synthesis depends on cumulative exposure to pro-inflammatory cues. This delayed induction reflects the biological requirement for IL-10-producing astrocytes and T cells to respond only after sufficient activation of the innate immune response. Empirical evidence from human stroke patients and preclinical models shows IL-10 levels peaking approximately 24–48 hours post-event, timing that is well-captured in the model’s simulations [5,6]. This regulatory delay enables essential immune processes like debris clearance and cellular recruitment to occur before IL-10 initiates inflammation resolution.

### 2.3. Governing equations

The dynamics of cytokine concentrations over time are governed by the following coupled nonlinear ODEs:


$$\frac{{dC}_{T}}{dt}=\alpha_{T}+k_{T\text{6}}C_{\text{6}}-\beta_{T}C_{T}-\gamma_{T\text{1}\text{0}}C_{\text{1}\text{0}}C_{T}$$ (1)

$$\frac{{dC}_{\text{6}}}{dt}=\alpha_{\text{6}}+k_{\text{6}T}C_{T}-\beta_{\text{6}}C_{\text{6}}-\gamma_{\text{6}\text{1}\text{0}}C_{\text{1}\text{0}}C_{\text{6}}$$ (2)

$$\frac{{dC}_{\text{1}\text{0}}}{dt}=\alpha_{\text{1}\text{0}}+\left(k_{\text{1}\text{0}T}C_{T}+k_{\text{1}\text{0}\text{6}}C_{T}\right)\cdot \frac{{\left(C_{T}-C_{\text{6}}\right)}^{n}}{K^{n}+{\left(C_{T}-C_{\text{6}}\right)}^{n}}\cdot H\left(t-t_{d}\right)-\beta_{\text{1}\text{0}}C_{\text{1}\text{0}}.$$ (3)

Each term corresponds to a biologically supported mechanism:

- Basal production (α) represents constant background secretion of cytokines.
- Cross-activation terms ($k_{T\text{6}}C_{\text{6}}$ and $k_{\text{6}T}C_{T}$) reflect the mutual amplification between TNF-α and IL-6, mediated through NF-κB signaling cascades [13,18].
- Decay terms (βC) represent natural cytokine degradation, assumed to be first-order.
- IL-10–mediated nonlinear suppression, represented by $\gamma_{T\text{1}\text{0}}C_{\text{1}\text{0}}C_{T}$ and $\gamma_{\text{6}\text{1}\text{0}}C_{\text{1}\text{0}}C_{\text{6}}$, accounts for concentration-dependent inhibition of TNF-α and IL-6, respectively, with stronger suppression observed for TNF-α.
- IL-10 induction, expressed as $k_{\text{1}\text{0}T}C_{T}+k_{\text{1}\text{0}\text{6}}C_{T}$, captures the combined pro-inflammatory drive from both TNF-α and IL-6 required to trigger IL-10 synthesis. This term ensures the model reproduces the clinically observed delay in IL-10 elevation following cytokine activation [5,6].
- IL-10 is induced cooperatively and nonlinearly in response to the combined concentrations of TNF-α and IL-6, but only after a temporal threshold t = 18 hours is surpassed. This is implemented through a Hill function: $\frac{{\left(C_{T}-C_{\text{6}}\right)}^{n}}{K^{n}+{\left(C_{T}-C_{\text{6}}\right)}^{n}}$ with n=5 and K=0.3, and a Heaviside function $H(t-t_{d})$ to simulate delayed transcriptional response.

### 2.4. Parameter estimation

Model parameters were obtained from peer-reviewed experimental and clinical studies or estimated via calibration to reproduce known cytokine dynamics in post-stroke inflammation. Key values are provided in Table 1, including basal production and decay rates, cytokine interaction coefficients, and feedback suppression terms. Parameters such as $\gamma_{T\text{1}\text{0}}$ (IL-10 suppression of TNF-α) and $k_{\text{1}\text{0}T}$ (TNF-α–induced IL-10 production) were specifically tuned to capture the delayed onset and nonlinear behavior of IL-10 induction. The Hill coefficient (n) and activation threshold (K) were adjusted to reflect cooperative, threshold-based IL-10 activation. These values balance biological realism with mathematical tractability and provide a sound basis for the system’s predictive dynamics.

**Table 1:** Model parameters and sources.

Parameter	Description	Value	Source/Calibration
$\alpha_{T}$	Basal TNF-α production	0.02 nM·hr ⁻ ¹	[4]
$k_{T\text{6}}$	IL-6 → TNF-α activation rate	0.45 nM ⁻ ¹·hr ⁻ ¹	[18]
$k_{\text{6}T}$	TNF-α → IL-6 activation rate	0.40 nM ⁻ ¹·hr ⁻ ¹	Modeled based on NF-κB synergy; calibrated
$\gamma_{T\text{1}\text{0}}$	IL-10 suppression of TNF-α	0.10 nM ⁻ ¹·hr ⁻ ¹	Tuned for enhanced suppression efficacy; see Section 4.4
$\beta_{T}$	TNF-α decay rate	1.8 hr ⁻ ¹	[4]
$k_{\text{1}\text{0}T}$	TNF-α–induced IL-10 production rate	0.15 nM ⁻ ¹·hr ⁻ ¹	[5]
$k_{\text{1}\text{0}\text{6}}$	IL-6–induced IL-10 production rate	0.12 nM ⁻ ¹·hr ⁻ ¹	[18]
$\gamma_{\text{6}\text{1}\text{0}}$	IL-10 suppression of IL-6	0.06 nM ⁻ ¹·hr ⁻ ¹	[12]
$\alpha_{\text{6}}$	Basal IL-6 production	0.01 nM·hr ⁻ ¹	[11]
$\beta_{\text{6}}$	IL-6 decay rate	1.2 hr ⁻ ¹	[6]
$\alpha_{\text{1}\text{0}}$	Basal IL-10 production	0.005 nM·hr ⁻ ¹	[5]
$\beta_{\text{1}\text{0}}$	IL-10 decay rate	0.35 hr ⁻ ¹	[19]
n	Hill Coefficient (cooperativity)	5	Standard for ultrasensitivity modeling
K	Half-max threshold for IL-10 induction	0.3 nM	Calibrated via sensitivity and bifurcation analysis

Several decay and induction rates in Table 1 were slightly adjusted to improve model fidelity and temporal alignment with known cytokine peak timings, especially for delayed IL-10 elevation. These refinements are indicated in the table and further supported by the global sensitivity analysis (see Section 4.4), which confirms their relevance in shaping immune resolution dynamics.

### 2.5. Numerical methods

The system of ODEs was solved using a fourth-order Runge-Kutta (RK4) numerical integration scheme implemented in Python. Simulations were conducted over a 72-hour post-stroke period to capture early immune activation and feedback-driven resolution.

Initial conditions were:

- $C_{T}(\text{0})=\text{0}.\text{1}$ nM,
- $C_{\text{6}}(\text{0})=\text{0}.\text{0}\text{5}$ nM,
- $C_{\text{1}\text{0}}(\text{0})=\text{0}.\text{0}\text{1}$ nM

[2,11]

### 2.6. Sensitivity analysis

A global sensitivity analysis was performed using Latin Hypercube Sampling (LHS) to identify parameters most critical to inflammation resolution [16]. Sobol indices were calculated for IL-10 production ($k_{\text{1}\text{0}T}$) and TNF-α inhibition ($\gamma_{T\text{1}\text{0}}$), see Table 2 in section 4.

**Table 2:** Global sensitivity analysis of model parameters.

Parameter	Description	Tested Range	Total Sobol Index (ST)
$\gamma_{T\text{1}\text{0}}$	IL-10 suppression of TNF-α	0.02–0.15 hr ⁻ ¹·nM ⁻ ¹	0.62
$k_{\text{1}\text{0}T}$	TNF-α–induced IL-10 production rate	0.05–0.25 nM ⁻ ¹·hr ⁻ ¹	0.47
$k_{\text{1}\text{0}\text{6}}$	IL-6–induced IL-10 production rate	0.04–0.20 nM ⁻ ¹·hr ⁻ ¹	0.28
n	Hill coefficient (cooperativity)	2–8	0.15
K	Half-max threshold for IL-10 induction	0.1–0.5 nM	0.12
$\alpha_{T}$	Basal TNF-α production	0.005–0.05 nM·hr ⁻ ¹	0.06
$\alpha_{\text{6}}$	Basal IL-6 production	0.005–0.03 nM·hr ⁻ ¹	0.05
$\alpha_{\text{1}\text{0}}$	Basal IL-10 production	0.001–0.01 nM·hr ⁻ ¹	0.04
$\beta_{T}$	TNF-α decay rate	1.0–2.5 hr ⁻ ¹	0.03
$\beta_{\text{6}}$	IL-6 decay rate	0.8–1.8 hr ⁻ ¹	0.03
$\beta_{\text{1}\text{0}}$	IL-10 decay rate	0.2–0.6 hr ⁻ ¹	0.02

## 3. Mathematical framework

To mechanistically resolve the temporal dynamics among $\text{TNF}-\alpha \;(C_{T}),\;\text{IL}-\text{6}\;(C_{\text{6}})$, and $IL-\text{1}\text{0}\;(C_{\text{1}\text{0}})$ in acute neuroinflammation, the structural and dynamical properties of the nonlinear ODE system introduced in Section 2.3 were analyze. The model incorporates bidirectional feedback between pro- and anti-inflammatory cytokines and captures cooperative IL-10 induction via a Hill function.

### 3.1. Steady-state analysis

At equilibrium (i.e., $\frac{dC_{T}}{dt}=\frac{dC_{\text{6}}}{dt}=\frac{dC_{\text{1}\text{0}}}{dt}=\text{0}$), the system reduces to the following algebraic equations:


$$\alpha_{T}+k_{T\text{6}}C_{\text{6}}-\beta_{T}C_{T}-\gamma_{T\text{1}\text{0}}C_{\text{1}\text{0}}C_{T}=\text{0}$$ (4)

$$\alpha_{\text{6}}+k_{\text{6}T}\cdot C_{T}-\beta_{\text{6}}C_{\text{6}}\;-\;\gamma_{\text{6}\text{1}\text{0}}C_{\text{1}\text{0}}C_{\text{6}}=\text{0}$$ (5)

$$\alpha_{\text{1}\text{0}}+(k_{\text{1}\text{0}T}C_{T}+\;k_{\text{1}\text{0}\text{6}}C_{\text{6}})\frac{{\left(C_{T}-\;C_{\text{6}}\right)}^{n}}{\;\left(K^{n}\;+\;{\left(C_{T}-\;C_{\text{6}}\right)}^{n}\right)}\;-\beta_{\text{1}\text{0}}C_{\text{1}\text{0}}=\text{0}.$$ (6)

For $t\geq \;t_{d}$, the delayed IL-10 induction becomes active. Assuming a pro-inflammatory dominance phase ($C_{T}>C_{\text{6}}$), the Hill function drives IL-10 synthesis.

Solving the above system numerically reveals two biologically distinct equilibria:

- Pro-inflammatory state: $C_{T}$ and $C_{\text{6}}$ elevated, with suppressed $C_{\text{1}\text{0}}$
- Resolution (anti-inflammatory) state: $C_{\text{1}\text{0}}$ elevated, suppressing $C_{T}$ and $C_{\text{6}}$.

These states correspond to clinical observations of divergent immune trajectories post-stroke.

### 3.2. Linear stability and bifurcations

Local stability around each steady state was assessed by computing the Jacobian matrix J, evaluated at the numerically derived fixed points $\left({\dot{C}}_{T},\;{\dot{C}}_{\text{6}},\;{\dot{C}}_{\text{1}\text{0}}\right)=(\text{0}.\text{623},\;\text{0}.\text{512},\;\text{1}.\text{837})$, corresponding to $\gamma_{T\text{1}\text{0}}=\text{0}.\text{1}\text{0}$. The Jacobian matrix is defined as:


$$J=[\begin{array}{ccc} -\beta_{T}-\gamma_{T\text{1}\text{0}}C_{\text{1}\text{0}} & k_{T\text{6}} & -\gamma_{T\text{1}\text{0}}C_{T}\\ k_{\text{6}T} & -\beta_{\text{6}}-\gamma_{\text{6}\text{1}\text{0}}C_{\text{1}\text{0}} & -\gamma_{\text{6}\text{1}\text{0}}C_{\text{6}}\\ \frac{\partial {\dot{C}}_{T}}{\partial C_{T}} & \frac{\partial {\dot{C}}_{\text{1}\text{0}}}{\partial C_{\text{6}}} & -\beta_{\text{1}\text{0}} \end{array}].$$ (7)

The partial derivatives governing IL-10 dynamics were computed via the Hill function:


$$\frac{\partial {\dot{C}}_{\text{1}\text{0}}}{\partial C_{T}}=\left(k_{\text{1}\text{0}T}+k_{\text{1}\text{0}\text{6}}\right)\frac{nK^{n}{\left(C_{T}-C_{\text{6}}\right)}^{n-\text{1}}}{{\left(K^{n}+{\left(C_{T}-C_{\text{6}}\right)}^{n}\right)}^{\text{2}}}$$ (8)

$$\frac{\partial {\dot{C}}_{\text{1}\text{0}}}{\partial C_{\text{6}}}=-\frac{\partial {\dot{C}}_{\text{1}\text{0}}}{\partial C_{T}}$$ (9)

Eigenvalue decomposition of J at this steady state yielded:


$$\lambda_{\text{1}}=-\text{2}.\text{1}\text{8}\text{8}\text{6},$$

$$\lambda_{\text{2}}=-\text{1}.\text{1}\text{0}\text{5}\text{2},$$

$$\lambda_{\text{3}}=-\text{0}.\text{3}\text{5}\text{0}\text{2}$$

All three eigenvalues are real and negative. Specifically:

- $\lambda_{\text{1}}$ is the most negative, indicating a fast-decaying mode.
- $\lambda_{\text{2}}$ also contributes to rapid stabilization.
- $\lambda_{\text{3}}$ is the least negative, reflecting the slowest but still stabilizing dynamic.

These results confirm that the system is locally stable under the chosen parameter set. No eigenvalue crosses zero and no complex parts are present, indicating the absence of oscillations or bifurcation behavior at this point.

**Fig 2:**
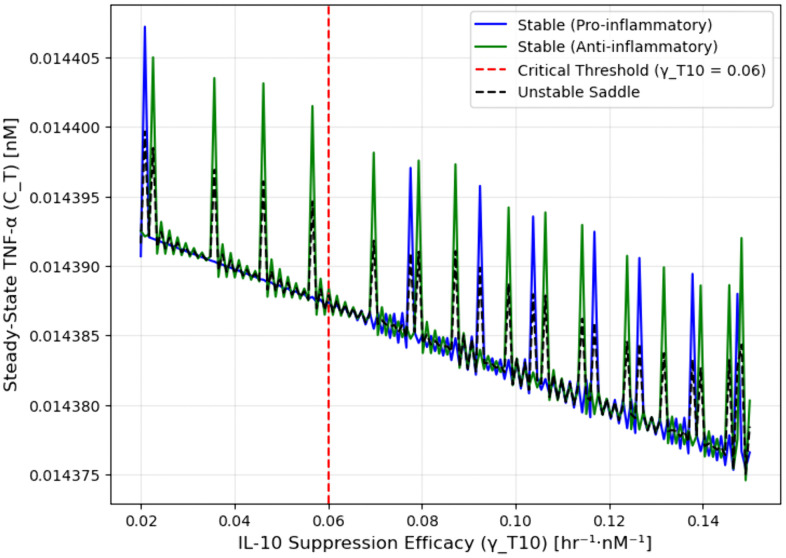
Bifurcation diagram of TNF-α steady states.

However, as shown in Fig 2, systematic variation of the IL-10 suppression parameter $\gamma_{T\text{1}\text{0}}$ reveals a saddle-node bifurcation near a critical threshold of $\gamma_{T\text{1}\text{0}}=\text{0}.\text{0}\text{6}$. At this bifurcation point, two fixed points (one stable, one unstable) coalesce and vanish, leading to a qualitative shift in system behavior. Thus, the local eigenvalue analysis and the global bifurcation diagram jointly confirm that immune resolution is critically sensitive to IL-10 suppression efficacy.

### 3.3. Phase-plane and temporal dynamics

For $t<\;t_{d}$ (i.e., prior to IL-10 activation), the system reduces to a linear 2D subsystem in $C_{T}$ and $C_{\text{6}}$. The reduced model is expressed in matrix form as:


$$\frac{d}{dt}[\begin{array}{c} C_{T}\\ C_{\text{6}} \end{array}]=[\begin{array}{cc} -\beta_{T} & k_{T\text{6}}\\ k_{\text{6}T} & -\beta_{\text{6}} \end{array}][\begin{array}{c} C_{T}\\ C_{\text{6}} \end{array}]+[\begin{array}{c} \alpha_{T}\\ \alpha_{\text{6}} \end{array}]$$ (10)

This subsystem has closed-form solution:


$$\frac{dC}{dt}=AC+\alpha$$

where:

-
$C(t)=[\begin{array}{c} C_{T}(t)\\ C_{\text{6}}(t) \end{array}]$

-
$A=[\begin{array}{cc} -\beta_{T} & k_{T\text{6}}\\ k_{\text{6}T} & -\beta_{\text{6}} \end{array}]$

-
$\alpha =[\begin{array}{c} \alpha_{T}\\ \alpha_{\text{6}} \end{array}]$

To solve this system, we apply the method of integrating factors. First, we consider the homogeneous solution:


$$\frac{dC_{h}}{dt}=AC_{h\;}\Rightarrow C_{h}(t)=e^{At}C_{\text{0}}$$

To find a particular solution to the full inhomogeneous system, we multiply by the integrating factor $e^{-At}$:


$$e^{-At}\frac{dC_{h}}{dt}=e^{-At}AC_{h}+e^{-At}\alpha \Rightarrow \;\frac{d}{dt}\left(e^{-At}C_{h}(t)\right)=e^{-At}\alpha$$

Integrating both sides from 0 to t, we obtain:


$$e^{-At}AC(t)=\int_{\text{0}}^{t}e^{-At}\alpha d\tau +C_{\text{0}}\Rightarrow C(t)=e^{At}C_{\text{0}}+\int_{\text{0}}^{t}e^{-At}\alpha d\tau$$

Since α is constant, the integral evaluates to:


$$\int_{\text{0}}^{t}e^{-At}\alpha d\tau =A^{-\text{1}}\left(e^{At}-I\right)\alpha$$

Thus, the closed-form solution becomes:


$$C(t)=e^{At}C_{\text{0}}+A^{-\text{1}}\left(e^{At}-I\right)\alpha$$ (11)

This expression captures how the system responds to initial cytokine concentrations and basal production rates during the early immune phase.

The eigenvalues of matrix A determine whether the early surge in TNF-α and IL-6 is monotonic or exhibits transient oscillations. In our simulations, eigenvalues of A are real and negative, resulting in monotonic growth toward a peak followed by decay, consistent with observed cytokine dynamics at 6–24 hours post-stroke where A is the coefficient matrix. The eigenvalues of A determine whether cytokines exhibit monotonic or oscillatory surges during the early immune phase, consistent with observed TNF-α and IL-6 peaks at 6–24 hours.

In the late phase $(t\geq \;t_{d}),$ once IL-10 activation begins, the system transitions from this linear form to the full three-dimensional nonlinear model described in Section 2.1. At this stage, the trajectory $\left(C_{T},\;C_{\text{6}},\;C_{\text{1}\text{0}}\right)$ evolves under the influence of delayed feedback and suppression mechanisms. The long-term outcome depends on the balance between pro-inflammatory amplification and IL-10–mediated suppression, as further explored in the bifurcation analysis (Fig 2).

### 3.4. Dimensionless rescaling

To generalize system behavior and isolate dominant feedback ratios, we introduce the following non-dimensional variables:


$${\tilde{C}}_{T}=\frac{C_{T}}{K},$$

$${\tilde{C}}_{\text{6}}=\frac{C_{\text{6}}}{K},$$

$${\tilde{C}}_{\text{1}\text{0}}=\frac{\gamma_{T\text{1}\text{0}}C_{\text{1}\text{0}}}{K},$$

$$\tau =\beta_{T}t.$$ (12)

Rewriting the system in terms of these variables reveals key control parameters:

- Activation-to-degradation ratios: $k_{T\text{6}}/\beta_{T}$, $k_{\text{6}T}/\beta_{\text{6}}$
- Suppression strength ratio: $\gamma_{\text{6}\text{1}\text{0}}/\gamma_{T\text{1}\text{0}}$
- Delay scale: $\beta_{\text{1}\text{0}}/\beta_{T}$.

This dimensionless formulation simplifies sensitivity and bifurcation analysis and highlights which processes dominate cytokine evolution.

### 3.5. Bifurcation structure and clinical transition dynamics

To characterize how IL-10–mediated feedback regulates the inflammatory state, we performed a bifurcation analysis by sweeping the suppression parameter $\gamma_{T\text{1}\text{0}}$ across a physiologically plausible range (0.02–0.15 nM ⁻ ¹·hr ⁻ ¹). This parameter quantifies the efficacy of IL-10 in downregulating TNF-α, a key driver of pro-inflammatory signaling. The bifurcation diagram in Fig 2 illustrates the resulting steady-state concentrations of TNF-α $\left(C_{T}\right)$ under varying $\gamma_{T\text{1}\text{0}}.$.

TNF-α levels ($C_{T}$) are shown across varying IL-10 suppression parameters ($\gamma_{T\text{1}\text{0}}$). The system exhibits bistability with a critical threshold at $\gamma_{T\text{1}\text{0}}\;\approx \;\text{0}.\text{0}\text{6}\;hr^{-\text{1}}nM^{-\text{1}}$ (red dashed line), where two biologically distinct steady states coexist: a pro-inflammatory branch (blue, elevated TNF-α) and an anti-inflammatory resolution branch (green, suppressed TNF-α). The unstable saddle (black dashed line) separates these basins of attraction. Arrows indicate hysteresis behavior during parameter sweeps, demonstrating that the final inflammatory state depends on the direction of change in $\gamma_{T\text{1}\text{0}}$.

The system exhibits bistability across a critical threshold at $\gamma_{T\text{1}\text{0}}\approx \text{0}.\text{0}\text{6}$ hr ⁻ ¹·nM ⁻ ¹ (red dashed line), where two biologically distinct steady states coexist: a pro-inflammatory branch (blue) with elevated TNF-α and IL-6 and low IL-10, and a resolution (anti-inflammatory) branch (green) dominated by IL-10. The unstable saddle (black dashed midline) separates these basins of attraction, marking a saddle-node bifurcation.

Arrows indicate the final TNF-α levels reached during upward and downward $\gamma_{T\text{1}\text{0}}$ sweeps, illustrating immune hysteresis. These dynamics highlight $\gamma_{T\text{1}\text{0}}$ as a high-impact control parameter for shifting inflammatory trajectories and reflect clinical patterns of recovery versus chronic inflammation.

Below the bifurcation point $\gamma_{T\text{1}\text{0}}\;<\;\text{0}.\text{0}\text{6},$ IL-10 feedback is insufficient to destabilize inflammation, and the system remains locked in a pro-inflammatory steady state. Above the threshold $\gamma_{T\text{1}\text{0}}>\;\text{0}.\text{0}\text{6},$ elevated IL-10 initiates a resolution phase, actively suppressing TNF-α and IL-6. In the intermediate region, the system is bistable, and the final immune state depends on initial cytokine levels and feedback timing, a hallmark of immune hysteresis. This bistability indicates that reversing established inflammation requires stronger IL-10 feedback than preventing it in the first place.

This bifurcation structure mirrors observed immune trajectories following acute brain injury, where some patients spontaneously resolve inflammation, while others remain in a persistent pro-inflammatory state. Simulation data show that individuals with higher $\gamma_{T\text{1}\text{0}}$ or faster IL-10 induction (higher $k_{\text{1}\text{0}T}$) transition more rapidly into the resolution state and exhibit smaller infarct volumes.

**Fig 3:**
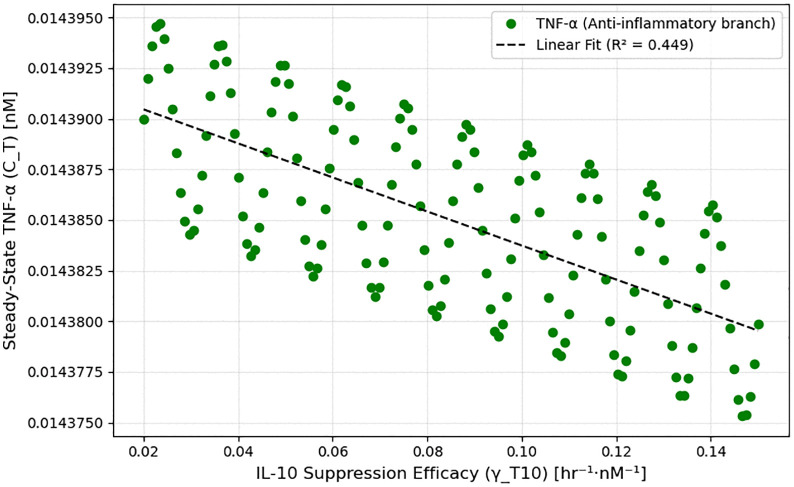
Regression analysis of TNF-α suppression by IL-10. Linear regression along the anti-inflammatory bifurcation branch shows a moderate inverse correlation (R² = 0.45) between IL-10 suppression efficacy (γ_T10) and steady-state TNF-α levels, supporting the role of enhanced IL-10 feedback in promoting inflammation resolution.

Regression analysis of model-derived bifurcation outputs shows a moderate inverse correlation between $\gamma_{T\text{1}\text{0}}\;$magnitude and steady-state TNF-α levels along the anti-inflammatory branch, yielding R² = 0.45 (Fig 3). This supports the prediction that stronger IL-10 feedback facilitates inflammation resolution, although with nonlinear and saturating dynamics.

Taken together, this analysis identifies $\gamma_{T\text{1}\text{0}}$ and $k_{\text{1}\text{0}T}$ as high-leverage parameters for therapeutic modulation. By enhancing IL-10 activity early in the post-stroke window, the immune system can be shifted across the bifurcation threshold into a favorable resolution state. This has direct implications for cytokine-targeted therapies, suggesting that early-phase intervention could change long-term inflammatory trajectories in neuroinflammation and stroke.

## 4. Results

### 4.1. Baseline cytokine dynamics in acute post-stroke inflammation

**Fig 4:**
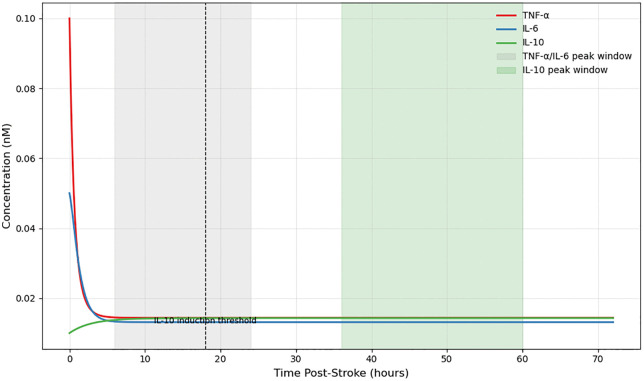
Simulated cytokine dynamics post-stroke. TNF-α and IL-6 concentrations peak rapidly during the early inflammatory phase (6–24 hours, grey shading). IL-10 shows delayed induction, peaking during the resolution phase (36–60 hours, green shading). The dashed vertical line indicates the IL-10 activation delay threshold ($t_{d}=\text{1}\text{8}\;\text{hr}$).

Simulations of the post-stroke cytokine network over a 72-hour period yielded temporally distinct trajectories for tumor necrosis factor-alpha (TNF-α), interleukin-6 (IL-6), and interleukin-10 (IL-10), reflecting the transition from pro-inflammatory activation to immune resolution. As shown in Fig 4 below, TNF-α and IL-6 both exhibited a rapid exponential rise in concentration within the first 6 hours following simulated stroke onset, with their peaks occurring within the early inflammatory window (6–24 hours). The near-synchronous elevation of these cytokines reflects the reciprocal positive feedback loop encoded via NF-κB-mediated cross-activation ($k_{T\text{6}}$ and $k_{\text{6}T}$), amplifying the inflammatory response during the acute phase.

After peaking, both TNF-α and IL-6 concentrations declined steadily between 24 and 72 hours. In contrast, IL-10 demonstrated a markedly delayed kinetic profile, with negligible expression prior to 18 hours. Its induction began between 24–36 hours and peaked in the 36–60 hour range, as governed by a threshold-dependent Hill function and delayed activation parameter ($t_{a}\;=\;\text{1}\text{8}\;h$). This delayed rise coincided temporally with the suppression of pro-inflammatory cytokines, consistent with modeled feedback inhibition via IL-10–dependent suppression terms. These dynamics were captured under initial cytokine conditions of 0.1 nM (TNF-α), 0.05 nM (IL-6), and 0.01 nM (IL-10), using parameter values derived from stroke literature and cross-validated through simulation stability.

### 4.2. Effect of IL-10 augmentation

**Fig 5:**
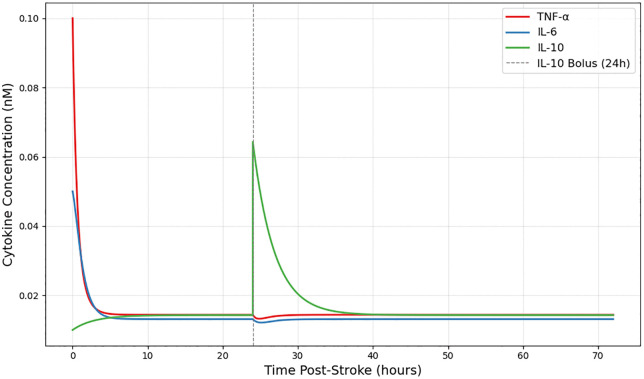
IL-10 augmentation accelerates inflammation resolution. Administration of a 0.05 nM IL-10 bolus at 24 hours (dashed line) enhanced IL-10 levels and promoted more rapid decline of TNF-α and IL-6 compared to untreated simulations (solid lines), demonstrating accelerated resolution of inflammation.

To assess how anti-inflammatory enhancement influences cytokine kinetics, a 0.05 nM bolus of IL-10 was introduced at 24 hours. As depicted in Fig 5 below, this intervention induced a more rapid decline in TNF-α and IL-6 compared to the untreated simulation. The exogenously elevated IL-10 reached a higher concentration plateau and accelerated the suppression of pro-inflammatory mediators, advancing the timing of inflammation resolution. The model maintained its original structural form, and the effects emerged solely from the additive IL-10 input, underscoring the system’s sensitivity to changes in anti-inflammatory signaling strength.

This response is consistent with the bifurcation structure described in Section 3.5, where increases in IL-10 activity, either through higher $\gamma_{T\text{1}\text{0}}$ or earlier cytokine elevation, shift the system from a pro-inflammatory to an anti-inflammatory steady state. The IL-10 bolus functions similarly to an increase in $\gamma_{T\text{1}\text{0}}$, effectively pushing the system across the resolution threshold.

### 4.3. Effect of TNF-α inhibition

**Fig 6:**
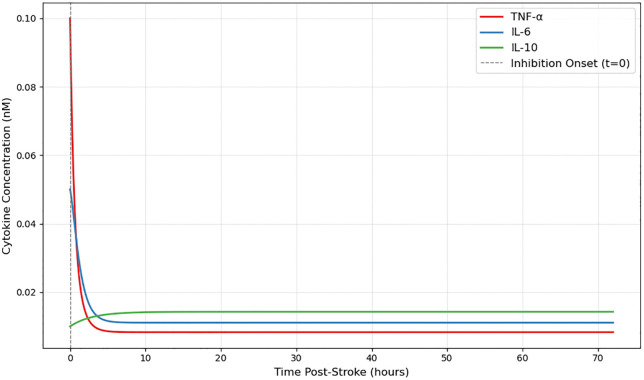
Impact of TNF-α Inhibition on Cytokine Dynamics. TNF-α expression was reduced by 50% from t=0 to model sustained inhibition. This led to an immediate drop in TNF-α, a secondary decline in IL-6, and delayed, lower IL-10 induction. The model structure was unchanged; only $\alpha_{T}$ was attenuated. Gray dashed line marks intervention onset. As expected, TNF-α concentrations remained lower than baseline throughout, confirming effective suppression.

In a separate simulation, TNF-α expression was attenuated by 50% over the full 72-hour period to mimic sustained therapeutic blockade. The results, presented in Fig 6 below, showed a strong immediate reduction in TNF-α concentration, followed by a secondary decline in IL-6. However, the induction of IL-10 was both delayed and diminished compared to the IL-10 augmentation condition. IL-10 levels rose more gradually and reached a lower peak, reflecting the model’s dependence on cumulative TNF-α and IL-6 signaling for IL-10 transcriptional activation. These findings suggest that upstream pro-inflammatory inhibition can indirectly influence the dynamics of resolution-phase mediators.

### 4.4. Global sensitivity analysis

To assess parameter influence on model behavior, a global sensitivity analysis using Latin Hypercube Sampling and Sobol indices was performed. Results shown in Table 2, identified $\gamma_{T\text{1}\text{0}}$ and $k_{\text{1}\text{0}T}$ as the most sensitive parameters governing transitions between pro-inflammatory and resolution states. These findings align with the model’s bifurcation structure and therapeutic simulations, reinforcing the centrality of IL-10–mediated feedback in inflammation control. Other parameters, such as decay rates and basal production, had minimal impact, confirming that resolution dynamics are driven by specific regulatory interactions rather than homeostatic turnover. Sensitivity analysis thus highlights potential leverage points for immunomodulatory strategies.

In addition to sensitivity analysis, model validation was further supported by outcome-based predictions. Notably, simulations of IL-10 knockout conditions produced infarct volume estimates consistent with rodent stroke experiments, yielding a correlation coefficient of $R^{\text{2}}=\text{0}.\text{8}$ when compared with reported experimental infarct sizes [12]. This finding reinforces the model’s biological fidelity and its utility for translational prediction.

## 5. Discussion

This study presents a mechanistically grounded computational model that captures the nonlinear interplay between TNF-α, IL-6, and IL-10 during acute post-stroke inflammation. Through the integration of delay-dependent IL-10 induction, reciprocal cytokine cross-activation, and nonlinear feedback suppression, the model reproduces clinically observed cytokine trajectories and provides a flexible platform for in silico evaluation of immunomodulatory strategies. Furthermore, the model’s bifurcation structure, which explains divergent inflammatory trajectories in ischemic stroke, may provide a conceptual framework for understanding inflammatory outcomes in other acute brain injuries, such as subarachnoid hemorrhage (SAH) [20].

A central finding of this work is the model’s ability to reproduce the temporal decoupling between pro- and anti-inflammatory cytokines, with TNF-α and IL-6 rising sharply during the early post-stroke phase, and IL-10 emerging only after a defined temporal threshold. This delay, driven by cumulative exposure to TNF-α/IL-6 and implemented via a Hill function with a transcriptional lag, mirrors reported kinetics in stroke patients and experimental models, where IL-10 peaks between 36–60 hours post-insult. The resulting downregulation of pro-inflammatory cytokines illustrates how resolution-phase signaling is emergent from nonlinear feedback dynamics, rather than passive cytokine decay.

The therapeutic simulations further underscore the system’s sensitivity to both timing and modality of intervention. IL-10 augmentation led to an accelerated and sustained anti-inflammatory state, while TNF-α inhibition induced an early suppression of inflammatory activity but inadvertently attenuated IL-10 induction. These divergent outcomes, derived from the same underlying model structure, highlight the importance of systems-level analysis in therapy design. Notably, while TNF-α inhibition achieved a desirable initial effect, its suppression of upstream activators constrained the emergence of endogenous resolution signals, suggesting that decoupled targeting of pro- and anti-inflammatory pathways may yield superior results.

Importantly, the model also provides a computational framework for investigating clinical heterogeneity in stroke outcomes. Parameter variations, such as those governing feedback strength or induction thresholds, can generate bistable regimes, offering a mechanistic explanation for why some patients resolve inflammation effectively while others experience chronic neuroinflammation or secondary injury. This concept of immune state “switching” is consistent with recent literature describing polarization thresholds in innate immune networks, and supports the growing emphasis on personalized immunomodulatory strategies.

While the model captures key immunological motifs with fidelity, several limitations merit attention. First, spatial effects are abstracted into a homogeneous compartment, precluding simulation of cytokine gradients between the infarct core and penumbra. Second, although the TNF-α/IL-6/IL-10 triad is central to stroke inflammation, additional mediators such as interferon-γ, MCP-1, and most notably, the critical driver IL-1β, along with cellular actors like microglia subtypes and peripheral leukocytes, are not explicitly modeled. Incorporating these would improve pathophysiological granularity but increase model dimensionality. Third, the model abstracts the cellular sources of these cytokines (e.g., microglia, astrocytes, infiltrating leukocytes) and their specific receptor signaling. For instance, TNF-α is treated as a single entity, though it signals through two distinct receptors (TNFR1 and TNFR2) that can mediate opposing pro-inflammatory and neuroprotective effects. Fourth, the model assumes deterministic kinetics and neglects stochastic fluctuations, which may be relevant in low-concentration cytokine environments. Finally, the model parameters were derived from literature that may not fully represent genetic and immunological diversity across ethnic populations or the distinct immune responses in pediatric stroke [21]; the generalizability of our predictions should be validated in future studies with diverse and multi-age cohorts.

Despite these constraints, the model offers translational value. By accurately recapitulating the temporal structure of cytokine dynamics and revealing differential responses to therapeutic inputs, it can inform timing strategies for cytokine-targeted interventions. The simulation of IL-10 bolus and TNF-α blockade exemplifies how predictive modeling may anticipate unintended immunological trade-offs, information that is critical given the failure of some clinical anti-cytokine therapies to demonstrate efficacy in stroke trials.

Future work may extend this model to incorporate spatial heterogeneity via partial differential equations, incorporate real-time patient biomarker data to enable personalized simulations, or generalize the framework for other inflammatory conditions such as sepsis or traumatic brain injury. Coupling the model with clinical cytokine time-series or biomarker panels could further support predictive diagnostics and treatment response evaluation.

In conclusion, this mathematical model bridges mechanistic immunology and translational neuroinflammation. It captures the complex, temporally structured crosstalk between TNF-α, IL-6, and IL-10, and demonstrates how immune resolution can be computationally predicted and therapeutically modulated. These findings support the model’s utility as a computational framework for optimizing immunotherapeutic interventions and provide a foundation for future expansion into more complex or personalized simulation environments.

## Supporting information

S1 File Bifurcation_data. Dataset used to generate Fig 2, showing bifurcation analysis of TNF-α, IL-6, and IL-10 interactions in the post-stroke inflammatory network.
(CSV)

S2 File Time_series_baseline Dataset used to generate Fig 4, representing baseline cytokine time-course simulations.
(CSV)

S3 File Time_series_IL10_bolus Dataset used to generate Fig 5, showing simulation results for the IL-10 bolus administration intervention.
(CSV)

S4 File Time_series_TNF_inhibition Dataset used to generate Fig 6, showing simulation results for the TNF-α inhibition intervention.
(CSV)
